# Voiding cystourethrography in patients undergoing endoscopic decompression of duplex system ureteroceles: to do or not to do?

**DOI:** 10.1007/s00383-024-05665-5

**Published:** 2024-04-10

**Authors:** Giorgia Contini, Ermelinda Mele, Andrea Celeste Barneschi, Ciro Esposito, Marco Castagnetti

**Affiliations:** 1 Division of Pediatric Urology, Bambino Gesù Children’s Hospital, IRCCS, Piazza Sant’Onofrio, 4, 00165 Rome, Italy; 2 Division of Pediatric Surgery, Federico II University of Naples, Naples, Italy; 3 Department of Surgery, Oncology, and Gastroenterology, University of Padova, Padua, Italy

**Keywords:** Ureterocele, Duplex system, Vesicoureteral reflux, Endoscopy, Urinary tract infection, Voiding cystourethrography

## Abstract

**Objective:**

To assess the role of voiding cystourethrography (VCUG) in patients with duplex system ureterocele (DSU) undergoing endoscopic decompression (ED).

**Materials and methods:**

This is a retrospective study of 75 consecutive patients with DSU undergoing ED [median (range) age, 6 (1–148) months]. Patients were divided into 3 groups, 33 with a VCUG showing vesicoureteral reflux (VUR) before ED (VUR-group), 22 with a VCUG negative for VUR (No-VUR-group), and 20 who did not undergo a VCUG (No-VCUG-group). Secondary surgery (SS) rate was compared among groups.

**Results:**

Groups were comparable for baseline characteristics. SS rate was 82% (27/33) in VUR-group vs. 32% (7/22) in the No-VUR-group (*p* = 0.0001), and 25% (5/20) in the No-VCUG-group (*p* = 0.001 vs. VUR-group, and 1 vs. No-VUR-group). In the VUR-group, 9 patients underwent preemptive endoscopic treatment of VUR during ED and SS rate was 44% (4/9) vs. 96% (23/24) in the remainder, *p*= 0.003. In the No-VCUG-group, a VCUG was performed during follow-up in 9/15 patients and showed reflux in all, although only 2 of these developed a (single) urinary tract infections.

**Conclusions:**

SS rate was significantly higher in patients with preoperative VUR. Instead, it was not significantly different between patients without VUR and those who did not undergo a VCUG before ED, despite all the latter who underwent a VCUG during follow-up had evidence of VUR generally in the absence of symptoms. In our opinion, a VCUG could be limited to patients developing symptoms after ED. If a VCUG is performed before ED, a preemptive treatment of VUR should be taken into consideration.

## Introduction

Vesicoureteral reflux (VUR) is common in duplex system ureterocele (DSU) patients occurring preoperatively in 50% to 75% of cases, more often in the lower ipsilateral moiety. VUR status may change following endoscopic decompression (ED) of the ureterocele, appearing de novo in any moiety or ceasing spontaneously [1–3].

Voiding cystourethrography (VCUG) is to date the gold-standard investigation to assess VUR, or its absence thereof in a patient with hydroureteronephrosis [4]. However, it is an invasive examination, both due to the X-rays exposure and the need for catheterization with the subsequent risks of urethral trauma and urinary tract infections (UTIs) [4].

For this reason, in patients with primary VUR, the role of VCUG has been widely reconsidered over recent years, and the prevalent attitude nowadays is to limit this investigation to selected patients [4–6].

VCUG is generally considered necessary in the workup of DSU patients [1]. To our knowledge, few studies have specifically focused on the role of this investigation in the decision-making and suggest that VCUG might be useful as the likelihood of ED to be effective as definitive treatment is inversely correlated to the number of renal units showing VUR on preoperative assessment [1, 7]. However, accumulating evidence has shown that VUR can resolve spontaneously during follow-up after ED [2, 3] and can be managed conservatively in asymptomatic and clinically stable patients [8, 9]. In keeping with this evidence, like for primary VUR [4–6], the actual role of VCUG in the management of DSU might be worth reconsidering.

Therefore, aim of the present study was to evaluate the role of VCUG in patients with DSU undergoing ED, both in terms of the need for it and the ideal timing in relation to ED, namely before or after ED. Our hypothesis was that a VCUG performed before ED could provide useful information for patient management and parental counseling.

## Materials and methods

After institutional ethics committee waiver, we retrospectively analyzed all consecutive DSU patients treated at our institution between January 2010 and December 2020. Only patients undergoing primary ED were included in this study. Patients undergoing other kinds of primary treatments, such as ureteroneocystostomy or (partial) nephrectomy, and those with associated urinary tract malformations, such as ureterocele in a solitary kidney, or with other associated malformations, such as syndromic patients, were excluded.

ED was performed in all patients under general anesthesia. The technique varied based on surgeon preference including either 1–2 punctures at the ureterocele base with the metallic guidewire of a 3 Fr ureteral catheter electrified with monopolar energy or multiple punctures with a Holmium laser fiber. A bladder catheter was left in place for 24 h after the procedure.

Patients included in the study were divided into three groups. The first group included patients undergoing preoperative VCUG which showed VUR in any moiety (VUR-group); second group included patients undergoing preoperative VCUG which showed no evidence of VUR (No-VUR-group); third group included patients who did not undergo a VCUG before ED (No-VCUG-group). VUR grade was classified as low when it was I or II and high grade when it was grade III or greater. Consistency among groups was assessed in terms of gender distribution, side of the ureterocele, history of prenatal diagnosis, preoperative history of febrile UTI, severity of upper urinary tract dilatation, and age at ED. The severity of upper urinary tract dilatation was expressed as antero-posterior diameter (APD) of the upper and lower pole pelvis on preoperative ultrasound (US).

Primary endpoint for the comparison of groups was the secondary surgery (SS) rate after primary ED, which included both VUR treatment (endoscopic or reimplantation) and ablative surgery (partial or total nephrectomy). Secondary endpoint included the rate of febrile UTIs or other symptoms after ED, and, in the No-VCUG-group, the results of follow-up VCUG, if performed. A sub-group analysis was performed in the VUR-group to compare patients who underwent preemptive endoscopic treatment of VUR at the same time of ED vs. those who did not.

The data were collected in an Excel file. Data were reported as median and range, or numbers and rates. Non-parametric tests were used for statistical analysis including the Kruskal–Wallis test for the comparison of unpaired continuous variables, and the Chi-square test or Fisher's exact test for categorical variables. SPSS software was used for statistical analysis. A *p *value < 0.05 was considered statistically significant.

## Results

**Fig. 1:**
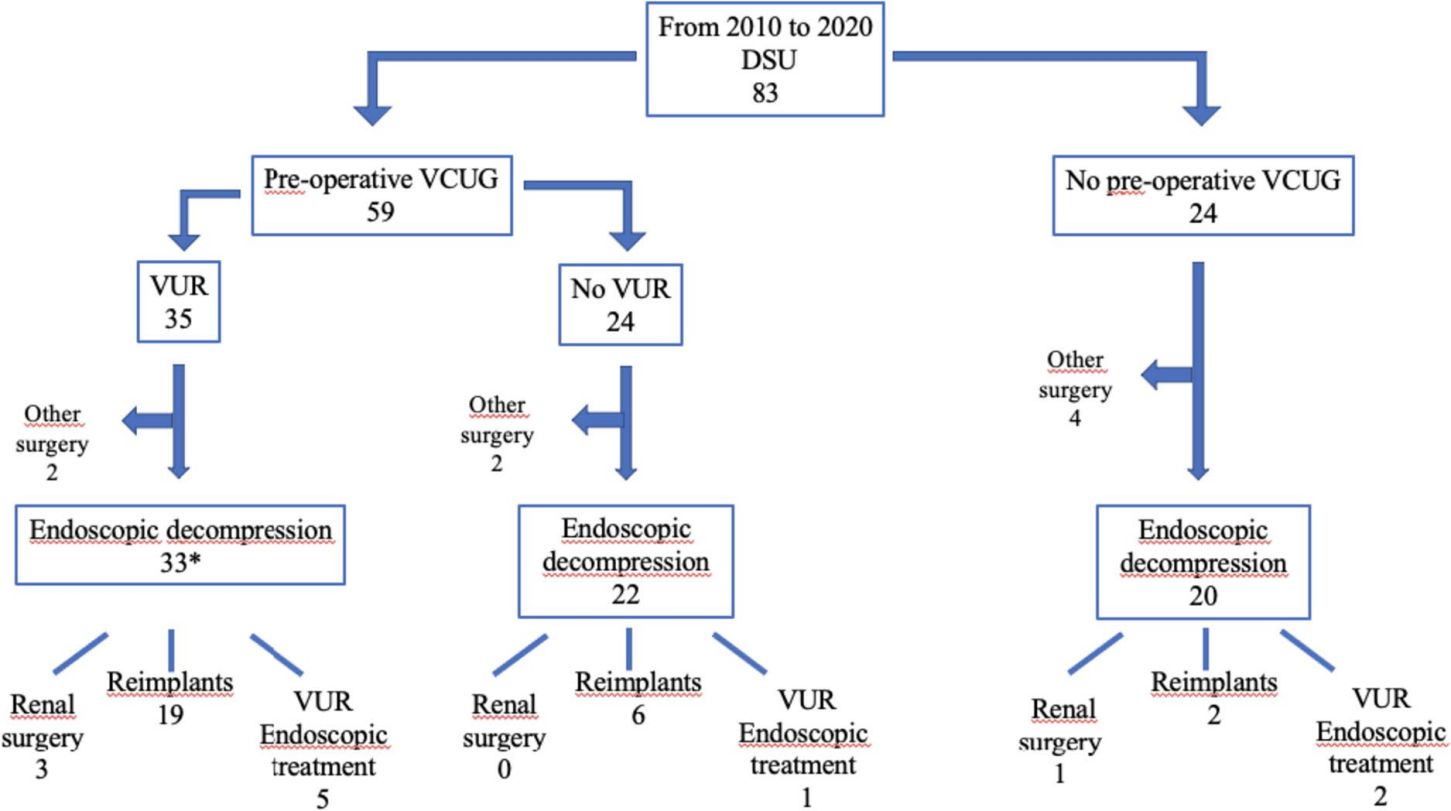
Flowchart showing the management in the study patients. *9 patients underwent preemptive endoscopic treatment of the VUR at the same time of ED.

We identified 83 DSU patients treated during the study period. Of these, 75 (90%) underwent primary ED at a median (range) age of 6 (1–148) months. Of the 75 patients, 33 (44%) were in the VUR-group, 22 (29%) in the No-VUR-group, and the remaining 20 (27%) in the No-VCUG-group (Fig. 1). Baseline characteristics were consistent among groups but for the history of prenatal diagnosis which was significantly less common in the VUR-group (Table 1).

**Table 1:** Baseline characteristics in the 3 groups

	VUR *n* = 33	NO VUR *n* = 22	NO VCUG *n* = 20	*p* value
Male gender n (%)	7 (21%)	7 (32%)	6 (30%)	0.6
Left side n (%)	18 (54%)	9 (41%)	10 (50%)	0.6
Prenatal diagnosis n (%)	12 (36%)	15 (68%)	13 (65%)	0.03
Preoperative UTIs n (%)	16 (57%)	11 (50%)	8 (40%)	0.7
AP diameter of the upper pole on preoperative US median (range) mm	17 (0–31)	12 (0–25)	15 (6–45)	0.4
AP diameter of the lower pole on preoperative US median (range) mm	5 (0–28)	1.5 (0–28)	3 (0–24)	0.7
Grade IV/V associated VUR				
Age at ED median [range] months	5 (1–144)	10 (1–120)	3.5 (1–148)	0.05
Technique for ED laser/monopolar incision				

In the VUR-group, VUR was high grade in 21, low grade in 5 and the grade was unknown in the remaining 7. Follow-up after ED was 57 (20–168) months, 67 (4–206) months, and 72 (6–135) months, respectively.

As summarized in Fig. 1 and Table 2, SS rate after ED was 82% (27/33), 32% (7/22), and 25% (5/20), respectively.

**Table 2:** Secondary surgery in the 3 groups

	VUR *n.* = 33	NO VUR *n* = 22	NO VCUG *n * = 20	*P* value
Secondary surgery n (%)	27 (82%)	7 (32%)	5 (25%)	0.00001
*Indications for secondary surgery*				
Febrile UTI	9	2	1	0.2
Persistent dilatation	3	2	1	
Asymptomatic VUR	15	2	1	
Lower urinary tract symptoms	0	1	2	
*Kinds of secondary surgery*				
Ureteroneocystostomy n (%)	19 (58%)	6 (27%)	2 (10%)	0.003
Endoscopic treatment of VUR n (%)	5 (15%)	1 (4%)	2 (10%)	0.4
Renal surgery n (%)	3 (9%)	0 (0%)	1 (5%)	0.3

Therefore, SS rate was significantly higher in the VUR-group compared to both the No-VUR (*p* = 0.0001) and No-VCUG-group (*p* = 0.001), whereas it was not statistically different between the latter two groups (*p* = 1). Of the patients undergoing surgery, 70% (16/23) in the VUR-group, 43% (3/7) in the No-VUR-group, and 40% (2/5) in the No-VCUG-group had no symptoms and the indication for SS was persistent VUR or lack of improvement of upper urinary tract dilatation.

In the VUR-group, 9 patients underwent preemptive endoscopic treatment of VUR at the same time of ED of the ureterocele, and 3 required ureteral reimplantation and another an additional endoscopic treatment of VUR during follow-up. Therefore, SS rate in these patients was 44% (4/9), which was significantly lower (*p* = 0.003) that the SS rate (96%, 23/24) in the remaining patients of the same group (Table 2). Grade of reflux was not different between groups, high and low grade 6 and 1 vs. 15 and 4, respectively, *p* = 1.

In the No-VCUG-group, 15/20 (75%) patients did not require any SS. A follow-up VCUG was performed despite the absence of UTI in 9 of these (60%) and showed VUR in all. VUR was high grade in 8 out of 9. During follow-up, 2 of these 9 patients developed a single UTI which did not recur afterwards (Table 2).

## Discussion

In present study, the SS rate was significantly higher in patients with a diagnosis of VUR before ED and particularly when a known VUR was not preemptively treated endoscopically at the same time of ED. SS rate was instead comparable in patients without VUR and in those who did not undergo a VCUG before ED. Of note, all of the patients without a preoperative VCUG who underwent a follow-up VCUG had evidence of VUR despite only 2 developed a single UTI during follow-up without further recurrences.

At first glance, the observation that SS rate was significantly higher in patients with preoperative VUR might seem confirming our testing hypothesis that a preoperative VCUG provides important information both for parental counseling and decision-making. Nevertheless, such a conclusion becomes questionable if considered in the context of the whole management strategy of DSU as outlined below.

Nowadays, a wide range of options is available for the primary treatment of DSU including conservative management, ED, upper pole partial nephrectomy, uretero-ureterostomy, and lower urinary tract reconstruction with or without concomitant upper pole partial nephrectomy [1]. ED has become increasingly popular over the last decades as primary treatment [10, 11]. However, its effectiveness as definitive treatment in DSU has been questioned, as studies suggested it is mainly a temporizing measure and allows for definitive treatment in less than half of the cases [12–15].

Nevertheless, the efficacy of a treatment critically depends on the expected goals and, therefore, the indications for SS. ED is unlikely to fully restore a normal functional anatomy, i.e., to achieve complete decompression of the upper urinary tract and resolution of VUR [1]. Therefore, if these are considered the goals of treatment, it is unlikely to be effective and the chance of success is inversely correlated to the number of renal units at jeopardy, i.e., showing VUR or non-refluxing hydroureteronephrosis before ED [7, 12]. In this framework, a preoperative VCUG would play an important role for risk-stratification and for the selection of the initial management. Nevertheless, evidence accumulating over the last 2 decades has questioned the role of VUR per se as an indication for SS. Jawdat et al. reported that among 14 patients who underwent SS for VUR after ED of ureterocele, only 4 presented with febrile UTI, while the remainder underwent surgery because of high-grade VUR or by parental request [8]. The same occurred in 70% of our patients in the VUR-group undergoing SS. Song et al. reported the presence of de novo VUR after ED in 59% of DSU, but such VUR remained often asymptomatic and was not associated with long-term deterioration of renal function [9]. Finally, Di Renzo et al. report that de novo VUR resulted in subsequent surgery in only 11% of their patients [17]. Moreover, other studies have documented the possibility of spontaneous resolution of VUR during post-ED follow-up. Jesus et al. observed VUR before ED in 40% of their patients, but the VUR resolved or improved spontaneously in 72% of cases during post-ED follow-up [2]. Similarly, Adorisio et al. reported spontaneous resolution of reflux in 13 of 19 patients (68%), in a group of 46 ureterocele patients undergoing ED [3].

In light of these data, we believe that the significantly higher SS rate in patients with preoperative VUR under many circumstances might reflect a proactive attitude of the surgeon in the detection and treatment of VUR rather than the treatment of a real clinical issue associated with the VUR detected by the investigation. Consistently, we consider far more relevant the fact that the SS rate in patients who did not undergo a preoperative VCUG was comparable to patients with no VUR on preoperative VCUG, despite 9 of 15 of the patients in the No-VCUG group had evidence of VUR on a follow-up VCUG and very seldom developed febrile UTIs and the latter did not recur.

If a proactive treatment of VUR is anyway considered appropriate, we think it noteworthy that the SS rate was significantly lower in our patients where the VUR detected on preoperative VCUG was treated endoscopically at the same time of ED vs. those where the VUR was left alone. Consistently, Kajbafzadeh et al. using the same strategy reported a SS rate of 10% after 4.2 years of follow-up, although this approach has not become a standard of care [18].

Although we believe that present data corroborate the principle that VUR after ED is not necessarily an indication for SS and that it does not need to be searched for systematically, we still believe paramount to use of an appropriate endoscopic technique for ED in order to minimize the risk to create a de novo VUR, especially in the decompressed ureterocele moiety [1, 19]. In this regard, we think it important to follow the recommendation to make a very dependent opening in the ureterocele, at the junction between the ureterocele and the bladder wall, regardless of the intravesical or ectopic location of the ureterocele, in order to create a flap mechanism after ED [20]. Creating a small opening is possibly another crucial factor to prevent VUR, although this also increases the risk for the procedure to fail decompressing the ureterocele satisfactorily [9, 19]. Use of laser fiber can be a useful adjunct to achieve good decompression with small and net punctures [20].

Overall, based on present experience and the available literature reported above, we believe that the role of VCUG in DSU patients could be reconsidered and, similarly to the practice currently used in primary VUR [4–6], the investigation could be limited only to patients developing febrile UTI or failing to show improvement in the hydroureteronephrosis after ED. Using these criteria, Castagnetti et al. reported a prevalence of VUR after ED of 32%, and a SS rate as low as 5% [16]. It should be noted also that the correlation between febrile UTI and VUR is not certain and should be considered cautiously in ureterocele patients, furthermore considering that the ureterocele can also be associated with a lower urinary tract dysfunction in some patients. Consistently, Singh and Smith noted that patients' tendency to develop UTI after ED of the ureterocele was not related to the presence of VUR. In their experience, about half of the patients (13/27) had VUR without ever presenting with UTI, while 3 had UTI without VUR [21].

Our study has limitations. Due to it retrospective design, some data are missing. The study size is somewhat small, although this is in line with the condition. Patient age range at treatment was wide, but median age was 6 months, which is consistent with common practice where ED is usually performed in infants. Results of renal nuclear scans were not considered. However, the function of ureterocele moieties is generally negligible and, in our practice, does not play a major role in the decision-making. Possibly most important, the technique used for ED as well as the indications for SS after ED were based on surgeon preference.

## Conclusions

In this retrospective study of patients with duplex system ureterocele undergoing endoscopic decompression, the secondary surgery rate was significantly higher in patients with vs. without vesicoureteral reflux on preoperative voiding cystourethrography. The secondary surgery rate was instead comparable in patients without vesicoureteral reflux and in those who did not undergo a voiding cystourethrography before endoscopic decompression, despite the fact that all the patients without a preoperative voiding cystourethrography who underwent a follow-up voiding cystourethrography had evidence of vesicoureteral reflux, generally asymptomatic.

In patients with preoperative evidence of vesicoureteral reflux, a preemptive endoscopic treatment of vesicoureteral reflux at the time of endoscopic decompression of the ureterocele decreased significantly the secondary surgery rate.

This study corroborates the hypothesis that a voiding cystourethrography might be limited to patients with symptoms or non-improving hydroureteronephrosis after endoscopic decompression.

## Data Availability

Anonymus data can be provided upon request to the corresponding author.

## References

[1] Castagnetti M, El-Ghoneimi A (2009) Management of duplex system ureteroceles in neonates and infants. Nat Rev Urol 6:307–315 19498409 10.1038/nrurol.2009.82

[2] Jesus LE, Farhat WA, Amarante AC, Dini RB, Leslie B (2011) Bägli DJ et al Clinical evolution of vesicoureteral reflux following endoscopic puncture in children with duplex system ureteroceles. J Urol 186:1455–1459 21862045 10.1016/j.juro.2011.05.057

[3] Adorisio O, Elia A, Landi L et al (2011) Effectiveness of primary endoscopic incision in treatment of ectopic ureterocele associated with duplex system. Urology 77(1):191–194. 10.1016/j.urology.2010.02.061 21168903 10.1016/j.urology.2010.02.061

[4] Arlen AM, Cooper CS (2019) New trends in voiding cystourethrography and vesicoureteral reflux: Who, when and how? Int J Urol 26(4):440–445. 10.1111/iju.13915 30762254 10.1111/iju.13915

[5] Johnin K, Kobayashi K, Tsuru T et al (2019) Pediatric voiding cystourethrography: an essential examination for urologists but a terrible experience for children. Int J Urol 26(2):160–171. 10.1111/iju.13881 30569659 10.1111/iju.13881

[6] Läckgren G, Cooper CS, Neveus T et al (2021) Management of vesicoureteral reflux: what have we learned over the last 20 years? Front Pediatr 31(9):650326. 10.3389/fped.2021.650326 10.3389/fped.2021.650326 PMC8044769 33869117

[7] DeFoor W, Minevich E, Tackett L et al (2003) Ectopic ureterocele: clinical application of classification based on renal unit jeopardy. J Urol 169:1092–1094. 10.1097/01.ju.0000049246.53911.04 12576859 10.1097/01.ju.0000049246.53911.04

[8] Jawdat J, Rotem S, Kocherov S et al (2018) Does endoscopic puncture of ureterocele provide not only an initial solution, but also a definitive treatment in all children? Over the 26 years of experience. Pediatr Surg Int 34:561–565. 10.1007/s00383-018-4258-9 29594460 10.1007/s00383-018-4258-9

[9] Song SH, Lee DH, Kim H et al (2019) Impact of de novo vesicoureteral reflux on transurethral surgery outcomes in pediatric patients with ureteroceles. Investig Clin Urol 60:295–302. 10.4111/icu.2019.60.4.295 31294139 10.4111/icu.2019.60.4.295 PMC6607067

[10] Byun E, Merguerian PA (2006) A meta-analysis of surgical practice patterns in the endoscopic management of ureteroceles. J Urol 176:1871–1877 16945677 10.1016/S0022-5347(06)00601-X

[11] Chertin B, De Caluwe D, Puri P (2003) Is primary endoscopic puncture of ureterocele a long-term effective procedure? J Pediatr Surg 38:116–119 12592632 10.1053/jpsu.2003.50023

[12] Husmann D, Strand B, Ewalt D et al (1999) Management of ectopic ureterocele associated with renal duplication: a comparison of partial nephrectomy and endoscopic decompression. J Urol 162:1406–1409 10492225

[13] Castagnetti M, Cimador M (2004) Sergio M et al Transurethral incision of duplex system ureteroceles in neonates: does it increase the need for secondary surgery in intravesical and ectopic cases? BJU Int 93:1313–1317 15180630 10.1111/j.1464-410X.2004.04861.x

[14] Park JS, Lee YS, Lee CN et al (2019) Transurethral incision as initial option in treatment guidelines for ectopic ureteroceles associated with duplex systems. World J Urol 37:2237–2244. 10.1007/s00345-018-2607-x 30603781 10.1007/s00345-018-2607-x

[15] Jain V, Agarwala S, Dhua A et al (2021) Management and outcomes of ureteroceles in children: an experience of 25 years. Indian J Urol 37:163–168. 10.4103/iju.IJU_522_20 34103800 10.4103/iju.IJU_522_20 PMC8173935

[16] Castagnetti M, Vidal E, Burei M et al (2013) Duplex system ureterocele in infants: should we reconsider the indications for secondary surgery after endoscopic puncture or partial nephrectomy? J Pediatr Urol 9:11–16. 10.1016/j.jpurol.2012.06.016 22819760 10.1016/j.jpurol.2012.06.016

[17] Di Renzo D, Ellsworth PI, Caldamone AA et al (2010) Transurethral puncture for ureterocele, which factors dictate outcomes? J Urol 184:1620–1624 20728127 10.1016/j.juro.2010.04.023

[18] Kajbafzadeh A, Salmasi AH, Payabvash S et al (2007) Evolution of endoscopic management of ectopic ureterocele: a new approach. J Urol 177:1118–1123. 10.1016/j.juro.2006.11.001 17296426 10.1016/j.juro.2006.11.001

[19] Castagnetti M, Capozza N (2022) Minimally invasive treatment of ureterocele. In: Esposito C, Subramaniam R, Varlet F, Masieri L (eds) Minimally Invasive Techniques in Pediatric Urology. Springer. 10.1007/978-3-030-99280-4_43

[20] Rich MA, Keating MA (1990) Snyder HM 3rd et al Low transurethral incision of single system intravesical ureteroceles in children. J Urol 144:120–121 2359157 10.1016/s0022-5347(17)39387-4

[21] Singh SJ, Smith G (2001) Effectiveness of primary endoscopic incision of ureteroceles. Pediatr Surg Int 17:528–531. 10.1007/s003830100586 11666051 10.1007/s003830100586

